# Weighting Bayesian information in human judgment under uncertainty: a hierarchical theory of cognitive biases

**DOI:** 10.3389/fpsyg.2026.1861515

**Published:** 2026-08-04

**Authors:** Bruno Kopp

**Affiliations:** Department of Neurology, Cognitive Neuropsychology, Hannover Medical School, Hannover, Germany

**Keywords:** Bayesian inference, cognitive biases, environmental uncertainty, evidence weighting, information integration, predictive coding, reliability learning, resource-rational cognition

## Abstract

Cognitive biases are typically viewed as departures from normative Bayesian reasoning. Instead, we propose that such biases emerge from a hierarchical cognitive architecture that adaptively regulates the weighting of prior beliefs and incoming evidence when cognitive resources are limited and uncertainty exists. While existing approaches, including heuristic, ecological rationality, Bayesian cognition, and resource-rational theories, capture important aspects of probabilistic inference, they differ in how inferential weighting is specified and adapted across contexts. We introduce Adaptive Bayesian Cognition (ABC), a hierarchical computational framework in which informational channels based on prior beliefs and evidence compete dynamically during belief updating. ABC distinguishes three interacting strata: first-order inferential updating in log-odds space; second-order reliability learning, which is driven by prediction error; and third-order adaptation to environmental uncertainty. Within this architecture, inferential weighting emerges from the competitive arbitration of informational sources based on attentional salience, cognitive cost, learned reliability, and inferred environmental uncertainty. ABC reconceptualizes cognitive biases as context-sensitive consequences of adaptive inferential arbitration, rather than as failures of rationality. Importantly, this framework makes a unique dynamical prediction: salience effects on evidence weighting should vary based on environmental uncertainty and learning history. This yields selective crossover dynamics that are not predicted by other accounts. Overall, ABC provides a unified computational framework that links probabilistic inference, reliability learning, and uncertainty adaptation in resource-constrained systems across ecological environments. Thus, ABC offers a computational framework of cognitive biases as systematic consequences of context-dependent evaluations of prior information and evidence in uncertain environments.

## 1 Human judgment under uncertainty: introduction

Research on human judgment and decision-making under uncertainty has long been shaped by competing interpretations of rationality (Tversky and Kahneman, 1974; Kahneman et al., 1982; Baron, 2023; Gilovich et al., 2002; Kahneman, 2003). The heuristics-and-biases tradition emphasizes systematic deviations from normative probabilistic reasoning. This tradition argues that human judgments frequently violate the principles of Bayesian inference by using heuristic shortcuts (Kahneman and Tversky, 1972, 1973). From this perspective, phenomena such as base-rate neglect, anchoring, and availability bias were interpreted as evidence that human cognition departs from probabilistic rationality (Kahneman and Tversky, 1972, 1973; Tversky and Kahneman, 1973; Bar-Hillel, 1980; Kahneman et al., 1982). In contrast, ecological-rationality approaches have argued that many heuristics are adaptive responses to environmental structures and computational limitations rather than irrational failures of inference (Brunswik, 1952; Todd and Gigerenzer, 2007; Gigerenzer and Brighton, 2009; Brighton and Gigerenzer, 2015). Furthermore, Bayesian models of cognition challenged the assumption that cognitive biases imply non-probabilistic reasoning. These models propose that human inference can remain approximately Bayesian, even when judgments appear distorted (Oaksford and Chater, 2007; Tenenbaum et al., 2011; Griffiths et al., 2024).

Together, these perspectives provide an incomplete picture of human rationality. If cognitive biases reflect violations of probabilistic inference, then human reasoning appears to be fundamentally non-Bayesian. However, if heuristics are ecologically adaptive, then many apparent biases may reflect context-sensitive strategies optimized for resource-constrained systems and uncertain environments. Bayesian approaches partially reconcile this tension by treating cognition as probabilistic inference under uncertainty. Yet, many existing Bayesian models are primarily normative and often leave unspecified how cognitive constraints dynamically shape the weighting of informational sources during inference. Consequently, a central theoretical question remains unresolved: Do cognitive biases indicate failures of Bayesian reasoning, or do they reflect adaptive distortions within an underlying probabilistic inference architecture?

The present article proposes Adaptive Bayesian Cognition (ABC; Kopp, 2025) as a hierarchical theory of Bayesian integration and cognitive biases in humans. This framework is based on the idea that human cognition involves the probabilistic integration of prior knowledge and incoming evidence. It also builds on existing Bayesian approaches by explicitly modeling how inferential weighting adapts under cognitive constraints, environmental uncertainty, and learning history. In ABC, cognitive biases are not considered isolated irrationalities or independent heuristic modules. Instead, they emerge from systematic shifts in the relative weighting of prior beliefs and incoming evidence during belief updating. Thus, the framework preserves the formal structure of Bayesian inference while incorporating ecological adaptation, bounded rationality, and resource-sensitive computation within a common inferential architecture (Brunswik, 1943, 1952; Simon, 1956; Todd and Gigerenzer, 2007; Lieder and Griffiths, 2020).

A central feature of the ABC framework is the distinction between normative Bayesian inference and adaptive subjective inference. Normative Bayesian models specify how beliefs about latent sates should be updated given prior probabilities and observed evidence. ABC instead proposes that human inference operates through adaptive arbitration between the two competing Bayesian informational sources whose relative influence changes dynamically as a function of attentional salience, cognitive cost, learned reliability, and environmental uncertainty. Importantly, the weighting parameter governing belief updating is not interpreted as a primitive psychological resource variable. Rather, inferential weighting emerges from competition between prior- and evidence-based utility signals that are themselves shaped by multiple upstream cognitive and environmental factors.

Within the ABC framework, several classic cognitive biases can be interpreted as different weighting regimes within a common inferential process. Base-rate neglect emerges when salient or vivid evidence dominates inferential updating at the expense of prior probabilities (Kahneman and Tversky, 1972, 1973; Bar-Hillel, 1980). Conservatism bias arises when previously accumulated beliefs maintain excessive influence despite incoming contradictory evidence (Phillips and Edwards, 1966; Peterson and Beach, 1967). Anchoring effects emerge when initial values acquire disproportionate inferential weight and persist across subsequent updates (Kahneman et al., 1982). Availability effects arise when highly accessible or salient information exerts amplified influence on evidence weighting (Tversky and Kahneman, 1973). Rather than reflecting qualitatively distinct violations of rationality, these phenomena are conceptualized as adaptive distortions arising from dynamic competition between Bayesian informational sources under constrained inference.

The present formulation is an extension of the earlier version of the ABC framework (Kopp, 2025), which introduces an explicitly hierarchical architecture composed of three interacting computational strata. The first stratum governs inferential belief updating through the weighted integration of priors and evidence. We refer to this as the core ABC model (see Section 2). The original contribution presented here is the explicit formulation of computational principles in the higher strata (see Section 3). Here, the second stratum governs experience-dependent learning of informational reliability through prediction error-based adaptation. The third stratum governs higher-order learning about the uncertainty structure of the environment. This hierarchical separation allows for a principled distinction between immediate belief dynamics, learning about informational trustworthiness, and adapting to long-term environmental uncertainty. Importantly, interactions across these strata generate emergent inferential dynamics that cannot be reduced to any single level in isolation.

This hierarchical organization enables ABC to produce empirically discriminative predictions that diverge from existing Bayesian, resource rational, and predictive processing frameworks (Alquier, 2020; Clark, 2013; Lieder and Griffiths, 2020; Parr et al., 2022; Butz et al., 2025). In particular, the model predicts that the influence of evidence salience on inferential weighting should evolve dynamically as a function of environmental uncertainty and accumulated learning history. In informative environments, salience initially amplifies evidence weighting, but it may later reduce it as stable predictive structures become dominant. Conversely, in noisy environments, salience effects are predicted to persist or increase over time. Thus, the framework predicts a distinctive interaction between salience, uncertainty, and temporal learning dynamics that could serve as a diagnostic marker distinguishing ABC from competing models of probabilistic inference.

The goal of this article is not to replace existing theories of attention, memory, or decision making with a single cognitive mechanism. Instead, ABC is presented as a hierarchical computational architecture that explains how various cognitive influences are incorporated into adaptive probabilistic inference. First, the article introduces the formal core ABC model of adaptive Bayesian integration (Section 2). Then, it develops the hierarchical extension involving reliability learning and uncertainty adaptation (Section 3). Finally, Section 4 situates the framework within major theoretical traditions related to the study of human judgment and decision-making in psychology and related fields. Section 5 provides an overview of individual differences in judgment and decision-making, as well as applied issues. The article also derives empirical predictions that distinguish the ABC framework from other theories of human inference. Overall, the article aims to provide a unified computational account of cognitive biases as emergent properties of adaptive Bayesian information integration under uncertainty.

## 2 Adaptive Bayesian cognition: the core framework

The core ABC framework, as originally introduced in Kopp (2025), proposes that cognitive biases originate from consistent differences in how individuals weigh prior knowledge and incoming evidence when updating their beliefs. Rather than viewing biases as violations of probabilistic reasoning, the ABC framework considers them context-dependent parameters within a single Bayesian inference architecture operating under cognitive resource constraints.

The formation of human beliefs about unobservable generative processes (or latent states) based on observable sensations can be conceptualized as the integration of two distinct sources of information. One source is retrieval-based knowledge, consisting of previously accumulated information stored in memory; this corresponds to prior beliefs in Bayesian inference. The second source is experience-based information, consisting of current sensory input or observed evidence about the environment, which corresponds to the likelihood component of Bayesian updating.

Bayesian inference (Jaynes, 2003) provides a normative account of how these information sources should be integrated under circumstances of uncertainty. For the purpose of this article, we consider a special case involving a discrete set of hypotheses about generative processes (e.g., a binary set $H=\{h_{1};h_{0}\}$ ). This is accomplished in the interest of simplicity; however, the line of reasoning can be extrapolated to other cases as well, including the continuous case. In accordance with the principles of Bayes’ rule, the one-step update of beliefs concerning a given hypothesis h (e.g., $h_{1}$ ) from a prior P(h) to a posterior P(h∣e) belief state is contingent upon the introduction of new evidence e .


$$P(h_{1}|e)=\frac{P(e|h_{1})\;P(h_{1})}{P(e|h_{1})\;P(h_{1})+P(e|h_{0})\;P(h_{0})}=\frac{P(e|h_{1})\;P(h_{1})}{P(e)\;}$$ (1)

In the context of one-step Bayesian belief updating in this binary case, the log-odds formulation is a convenient representation (Lindley, 1985). The multiplicative Bayes’ rule (Equation 1) is here transformed into a simple additive process when the logarithm of the odds ratio, henceforth referred to as LO , is taken. The LO of the probabilities for $h_{1}$ and $h_{0}$ is calculated as:


$$\log\frac{P(h_{1}|e)}{P(h_{0}|e)}=\log\;\frac{P(h_{1})}{P(h_{0})}+\log\;\frac{P(e|h_{1})}{P(e|h_{0})}$$ (2)

This formulation elucidates how the log-posterior odds $({\mathrm{LO}}_{\text{posterior}}\;$ = log $\frac{P(h_{1}|e)}{P(h_{0}|e)}$ ) are simply determined as the summation of the log-prior odds $({\mathrm{LO}}_{\text{prior}}\;$ = log $\frac{P(h_{1})}{P(h_{0})}$ ) and the log-likelihood ratio ( ${\mathrm{LLR}}_{e}$ = log $\frac{P(e|h_{1})\;}{P(e|h_{0})\;}$ ).

The process of normative Bayesian updating integrates prior knowledge and new information without distortion, as delineated in Equations 1, 2. However, a substantial corpus of empirical research demonstrates that human judgments frequently deviate systematically from Bayes’ rule. Classic examples include conservatism, defined as an inadequate adjustment to new evidence (Phillips and Edwards, 1966; Peterson and Beach, 1967), and base-rate neglect, defined as an inadequate use of prior knowledge during belief revision (Kahneman and Tversky, 1972, 1973; Bar-Hillel, 1980). These phenomena are generally regarded as failures of probabilistic reasoning or consequences of heuristic shortcuts (Kahneman et al., 1982).

The ABC framework offers an alternative interpretation. Contrary to the assumption that people do not apply Bayesian reasoning, the ABC framework posits that individuals engage in *weighted* Bayesian updating. In this process, the relative influence of priors and evidence is modulated by cognitive resource constraints, attentional salience, and reliability estimation, as discussed in the subsequent section.

According to the ABC framework (Equation 3), in the special case of a binary set of generative hypotheses, subjective one-step belief updating is formalized as a weighted transformation from normative log-prior odds to subjective log-posterior odds:


$${\hat{\mathrm{LO}}}_{\text{posterior}}=\gamma \;{\mathrm{LO}}_{\text{prior}}+(1-\gamma )\;{\mathrm{LLR}}_{e}$$ (3)

In this context, the parameter γ∈[0,1] denotes the relative weight assigned to prior beliefs, while 1−γ specifies the weight assigned to current evidence. The constraint that weights sum to one ( γ+(1−γ)=1 ) reflects a fundamental resource limitation: cognitive systems must allocate finite processing capacity across competing informational sources.

Conceptually, γ is closely related to probability weighting functions in decision theory, which describe systematic deviations between objective probabilities and their subjective representation (Tversky and Kahneman, 1992; Zhang and Maloney, 2012). Within the ABC framework, such distortions are not applied to probabilities per se, but rather to the integration of priors and likelihoods during belief updating. In this manner, the model maintains the structure of Bayesian inference while permitting systematic variation in the allocation of cognitive resources to informational sources (Fiedler and Juslin, 2006; Juslin et al., 2008; Fennell and Baddeley, 2012; Juslin, 2015). This formulation aligns with resource-rational approaches, which interpret deviations from normative inference as adaptive responses to computational constraints (Simon, 1956; Gershman et al., 2015; Lieder and Griffiths, 2020). The weighting parameter γ thus provides a computational link between probabilistic inference and cognitive resource allocation.

Following this line of reasoning, cognitive biases can be conceptualized as different operating modes within a shared inferential framework. The weighting parameter, γ , defines the relative influence of prior beliefs and incoming evidence during belief updating. As γ approaches 0.5, the updating process approximates balanced Bayesian integration, though it does not achieve exact optimality (Kopp, 2025). Values above 0.5 reflect prior-dominant updating, wherein existing beliefs constrain inference disproportionately. Values below 0.5 reflect evidence-dominant updating, wherein incoming evidence overrides prior structure. Importantly, ABC interprets these deviations as adaptive consequences of resource-constrained inference under varying environmental conditions, not as irrational errors (Simon, 1956; Lieder and Griffiths, 2020).

According to the ABC framework, seemingly distinct cognitive biases are expressions of different weighting regimes rather than separate psychological mechanisms. Prior-dominant regimes explain conservatism (Phillips and Edwards, 1966; Peterson and Beach, 1967), anchoring (Tversky and Kahneman, 1974; Kahneman et al., 1982; Lieder et al., 2018), confirmation bias (Klayman, 1995; Pilgrim et al., 2024), belief perseverance (Anderson, 2007), and status quo bias (Samuelson and Zeckhauser, 1988). All of these involve an excessive reliance on accumulated knowledge or initial beliefs. Evidence-dominant regimes, on the other hand, account for base-rate neglect and representativeness (Kahneman and Tversky, 1972, 1973; Bar-Hillel, 1980), availability effects (Tversky and Kahneman, 1973), and jumping-to-conclusions biases (Garety et al., 2011; Ross et al., 2015). These biases involve the disproportionate influence of salient or vivid evidence on inference. Thus, these biases reflect systematic distortions within a shared Bayesian updating process rather than distinct failures of rationality.

ABC also aligns with Savage's (1954) distinction between small-world and large-world environments. Structured statistical tasks with explicit priors tend to promote prior-dominant updating, which often produces conservatism effects (Phillips and Edwards, 1966; Peterson and Beach, 1967). In contrast, uncertain causal environments containing vivid or case-specific evidence, such as the cab problem, tend to promote evidence-dominant updating and base-rate neglect (Kahneman and Tversky, 1972, 1973; Bar-Hillel, 1980). Thus, the ABC framework can explain why different experimental paradigms produce different biases (see Table 1) without positing distinct mechanisms for each phenomenon (Kopp, 2025).

**Table 1:** Summary of the unified weighting interpretation of major cognitive biases.

Weighting regime	Inferential profile	Representative biases
γ ≈ 0.5	Approximate Bayesian integration	Near-normative reasoning
γ > 0.5	Prior-dominant updating (small world)	Conservatism, anchoring, confirmation bias, belief perseverance, status quo bias
γ < 0.5	Evidence-dominant updating (large world)	Base-rate neglect, representativeness, availability, jumping-to-conclusions
Extreme γ values	Single-source reliance under high constraints	Heuristic responding under very high cognitive load

Under conditions of very high cognitive load or severe informational constraints, inferential weighting may approach extreme values ( γ → 0 or γ → 1). This results in a nearly exclusive reliance on a single informational source. In these situations, cognition shifts from weighted integration to heuristic responding because maintaining balanced probabilistic updating becomes computationally infeasible (Simon, 1956; Kahneman, 2003). Extreme prior-dominant regimes may produce rigid belief persistence despite contradictory evidence. In contrast, extreme evidence-dominant regimes may produce highly reactive judgments driven by immediately salient inputs. From an ABC perspective, these patterns represent adaptive simplifications of inference under severe capacity limitations rather than distinct reasoning systems.

**Figure 1:**
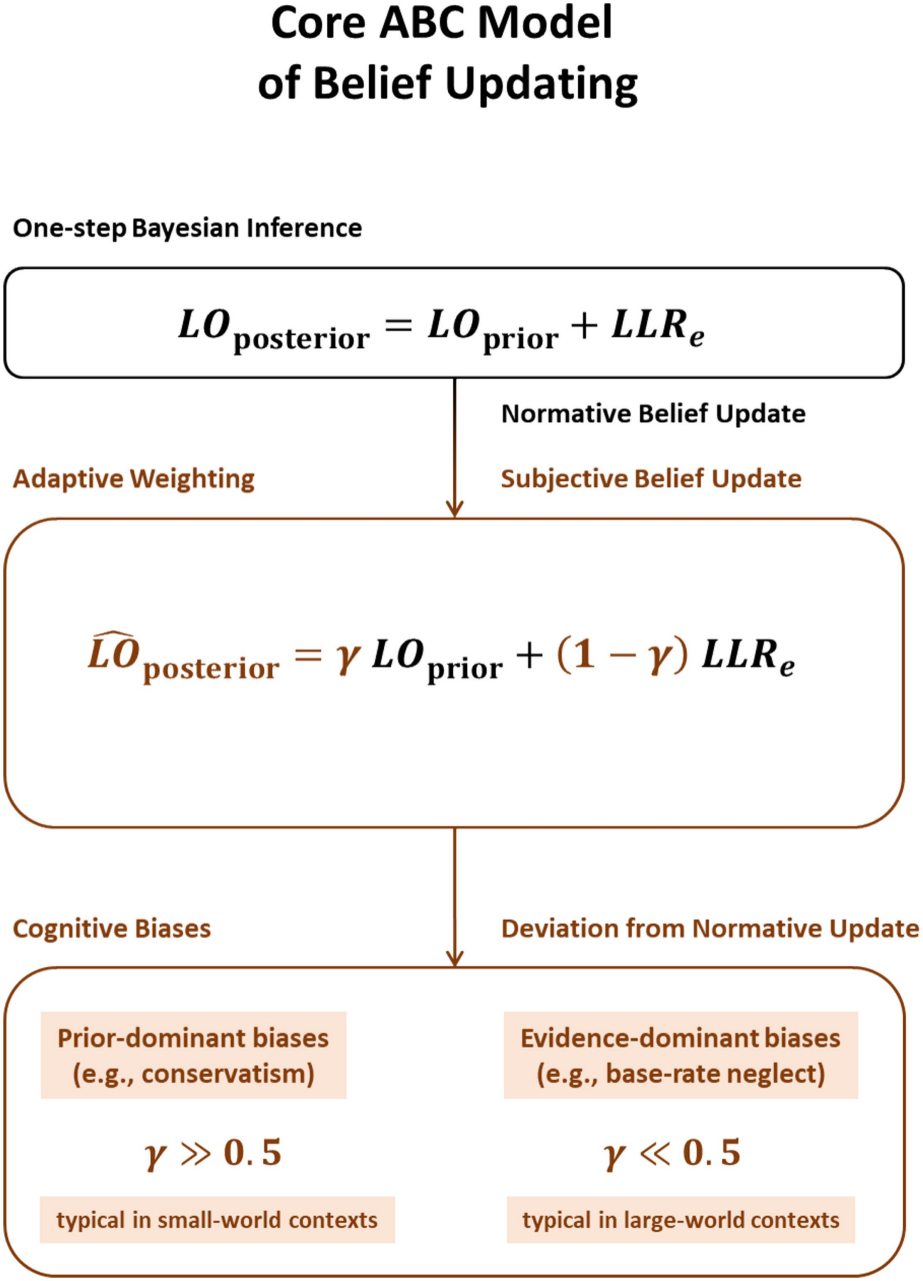
The ABC model describes cognitive biases as adaptive Bayesian updating under resource constraints. Prior weighting is represented by γ∈ [0,1] and evidence weighting is represented by (1−γ) . Cognitive biases arise from context-sensitive probability weighting, which is driven by variations in task context. The specific context is determined by the transparency of priors and cognitive cost related to their retrieval, the salience of case-specific evidence, and the estimated reliability of information. This process enables boundedly rational agents to efficiently allocate their limited cognitive resources across informational cues. Context-sensitive γ regimes result in biases: rior overweighting (conservatism) when γ≫0.5 and evidence overweighting (base-rate neglect) when γ≪0.5 . Approximate, though not equivalent, normative Bayesian beliefs are formed only when γ≈0.5 .

Overall, the unified ABC formulation replaces a diverse set of explanations for specific biases with a hierarchical computational model. In this model, cognitive biases emerge from adaptive shifts in inferential weighting under uncertainty. Figure 1 provides a synopsis of the core ABC framework, offering an innovative interpretation of cognitive biases as parameter-dependent regimes within a cohesive Bayesian architecture. Instead of indicating failures of rationality, biases emerge from systematic, context-sensitive reallocations of limited cognitive resources. This perspective integrates the heuristics-and-biases tradition, ecological rationality, Bayesian cognition, and resource-rational theory within a single formal model.

Consequently, the central question shifts from whether human reasoning is Bayesian to the conditions under which the weighting of priors and evidence changes. Cognitive biases are thus best understood as adaptive modes of probabilistic inference operating under resource constraints, rather than as violations of normative principles.

## 3 Adaptive Bayesian cognition: hierarchical extension

The ABC framework posits that cognitive biases stem from systematic differences in how individuals weigh prior knowledge and incoming evidence when updating their beliefs. Instead of representing violations of probabilistic reasoning, biases reflect deviations within a Bayesian inference architecture that operates under cognitive resource constraints and contextual influences. The framework distinguishes between normative Bayesian inference, which specifies optimal belief updating according to Bayes’s rule, and subjective inference, which reflects the adaptive updating of beliefs by a cognitive system with limited resources. Human belief formation integrates two types of information: retrieval-based knowledge from memory (corresponding to prior beliefs) and experience-based knowledge from current sensory evidence (corresponding to likelihood information).

Sequential Bayesian inference in log-odds form yields an additive update rule (Equation 4). The posterior belief at time t equals the prior belief at time t−1 plus the log-likelihood ratio of the evidence at time t . Denoting the normative Bayesian belief trajectory as ${\mathrm{LO}}_{t}$ , the recursive Bayesian update is given by:


$${\mathrm{LO}}_{t}={\mathrm{LO}}_{t-1}+{\mathrm{LLR}}_{e_{t}}$$ (4)

In contrast, the hierarchical ABC framework assumes that subjective belief updating follows a weighted integration rule (Equation 5).


$${\hat{\mathrm{LO}}}_{t}=\gamma_{t}\;{\hat{\mathrm{LO}}}_{t-1}+(1-\gamma_{t})\;{\mathrm{LLR}}_{e_{t}}$$ (5)

where ${\hat{\mathrm{LO}}}_{t-1}$ denotes the subjective prior belief state, $\;\gamma_{t}\in$ [0,1] represents the weight assigned to prior beliefs at time t , and $\left(1-\gamma_{t}\right)$ denotes the weight assigned to the current evidence quantified as the likelihood information it provides, ${\mathrm{LLR}}_{e_{t}}$ . Initially, subjective and normative beliefs are assumed to coincide $\left({\hat{\mathrm{LO}}}_{0}={\mathrm{LO}}_{0}\right)$ , such that deviations from the Bayesian solution emerge over time as a function of the weighting parameter $\;\gamma_{t}$ . Thus, cognitive biases can be understood as systematic divergences between subjective and normative belief trajectories rather than failures to apply probabilistic principles.

### 3.1 Dual-source utility model of epistemic valuation

We propose a dual-source utility formulation (Equations 6a, 6b, 7, 8a, 8b) in which inferential weighting arises from the interaction between prior- and evidence-based valuation systems. Each utility component is defined by distinct epistemic inputs that contribute to Bayesian belief updating, yet they remain functionally separable at the representational level.

We assume that the prior-based utility, $u_{{\text{prior}}_{t}}$ , depends on the estimated reliability of prior knowledge, $r_{{\text{prior}}_{t}}$ , and the cognitive costs associated with retrieving and maintaining accumulated information from memory, $C_{t}$ .


$$u_{{\text{prior}}_{t}}=f(r_{{\text{prior}}_{t}},C_{t})$$ (6a)

where utility increases with estimated prior reliability and decreases with cognitive costs:


$$\frac{\partial u_{{\text{prior}}_{t}}}{\partial r_{{\text{prior}}_{t}}}>0\;\text{and}\frac{\partial u_{{\text{prior}}_{t}}}{\partial C_{t}}<0.$$

The evidence-based utility, $u_{{\text{evidence}}_{t}}$ , depends on the estimated reliability, $r_{{\text{evidence}}_{t}}$ , and attentional salience, $S_{t}$ , of incoming information.


$$u_{{\text{evidence}}_{t}}=f(r_{{\text{evidence}}_{t}},S_{t})$$ (6b)

with utility increasing as a function of both evidence reliability and salience:


$$\frac{\partial u_{{\text{evidence}}_{t}}}{\partial r_{{\text{evidence}}_{t}}}>0\;\text{and}\;\frac{\partial u_{{\text{evidence}}_{t}}}{\partial S_{t}}>0.$$

Both utility functions are assumed to exhibit diminishing marginal returns as reliability and salience increase.

The inferential prior weighting is determined by the relative epistemic strength of these competing utility signals:


$$\gamma_{t}=\frac{u_{{\text{prior}}_{t}}}{u_{{\text{prior}}_{t}}+u_{{\text{evidence}}_{t}}}$$ (7)

with (1 − $\gamma_{t}$ ) determining the evidence weighting correspondingly. This formulation specifies a competitive arbitration mechanism in which the weighting is endogenous, context-sensitive, and dynamically determined by the relative utility of the prior and evidence channels, rather than by fixed inferential parameters.

The model yields three limiting regimes. When prior utility dominates evidence utility ( $\gamma_{t}$ → 1), prior-dominant updating occurs. When evidence utility dominates ( $\gamma_{t}$ → 0), evidence-dominant updating occurs. When utility is approximately equal, symmetric arbitration occurs ( $\gamma_{t}$ ≈ 0.5).

A tractable instantiation of the model can be written as:


$$u_{{\text{prior}}_{t}}=a\;\log\;(r_{{\text{prior}}_{t}})-b\;C_{t}\;$$ (8a)

and


$$u_{{\text{evidence}}_{t}}=c\;\log\;(r_{{\text{evidence}}_{t}})+d\;\log\;(S_{t})$$ (8b)

where *a*, *b*, *c*, and *d* are positive scaling parameters. The logarithmic specification captures diminishing marginal sensitivity to reliability and salience. In contrast, cognitive costs impose a linear penalty on memory-based processing. This asymmetry reflects the assumption that internally retrieved information incurs computational costs not associated with immediate sensory evidence.

Together, these equations formalize inferential weighting as a competition between epistemic valuation systems operating on prior knowledge and incoming evidence. As shown below, these utilities are dynamically recalibrated through reliability learning driven by prediction error and higher-order uncertainty adaptation within the hierarchical ABC framework. This links inferential weighting to cognitive cost, attentional allocation, learning history, and environmental structure.

### 3.2 Hierarchical reliability and uncertainty learning in adaptive Bayesian cognition

A central implication of the hierarchical ABC framework is that inferential weighting is adaptive and experience-dependent, rather than fixed. The model distinguishes three interacting levels of computation: first-order belief updating, second-order reliability learning, and third-order estimation of environmental uncertainty. These levels determine how priors and incoming evidence are weighted over time.

At the first level, beliefs are represented and updated in log-odds ( LO ) space through weighted integration of prior beliefs and incoming evidence as specified in Equation 5. Predictions about future observations (Equations 9, 10) are generated from the current belief state in probability space:


$$\hat{P}{\left(h_{1}\right)}_{t-1}=\sigma ({\hat{\mathrm{LO}}}_{t-1})\;\;\text{and}\;\hat{P}{\left(h_{0}\right)}_{t-1}=1-\hat{P}{\left(h_{1}\right)}_{t-1}$$ (9)

with σ denoting the logistic transformation. Expected evidence is then computed as:


$$\hat{P}(e_{t})=P(e_{t}|h_{1})\;\hat{P}{\left(h_{1}\right)}_{t-1}+P(e_{t}|h_{0})\;\hat{P}{\left(h_{0}\right)}_{t-1}$$ (10)

Prediction errors arise from discrepancies between predicted and realized outcomes (Equation 11) and drive higher-order learning processes:


$$\delta_{t}=\kappa_{t}-|I(e_{t})-\hat{P}(e_{t})|$$ (11)

where $I(e_{t})\in \{0,1\}$ indicates the realized outcome and $\kappa_{t}\in [0,1]$ denotes the estimated environmental uncertainty. Importantly, prediction errors do not update beliefs. Rather, they update the inferred reliability r∈[0,1] of informational sources:


$$\;r_{t+1}=f(\;r_{t},\delta_{t})$$ (12)

Reliability learning (Equation 12) therefore operates as a second-order process governing subsequent inferential weighting.

The third stratum involves learning about environmental uncertainty itself. In the current framework, $\kappa_{t}$ reflects the estimated predictability of the environment throughout the learning process. Low values of $\kappa_{t}$ correspond to stable and informative environments, where prediction errors are diagnostic and a reliable predictive structure can be learned efficiently. Conversely, high values of $\kappa_{t}$ correspond to noisy or weakly informative environments, where prediction errors remain ambiguous, and stable predictive structures are difficult to establish.

Under the binary restriction, the hierarchical ABC architecture can be factorized into three coupled computational strata. At the first stratum, beliefs are updated in log-odds ( LO ) space through weighted integration of prior beliefs and incoming evidence. Incoming evidence is represented by the log-likelihood ratio ${\mathrm{LLR}}_{e_{t}}$ , such that larger absolute ${\mathrm{LLR}}_{e_{t}}$ values correspond to more diagnostically informative observations. This inferential process maps evidence onto posterior beliefs, whereas prediction maps current beliefs onto expected observations.

At the second stratum, prediction errors drive adaptive learning of the inferred reliability of prior- and evidence-based informational sources rather than directly modifying beliefs themselves. At the third stratum, the system estimates environmental uncertainty, indexed by $\kappa_{t}$ , which governs the long-run stability properties of reliability learning. Informative environments characterized by stable and discriminative likelihood structure progressively reduce estimated uncertainty, whereas noisy environments with weak or inconsistent likelihood ratios maintain elevated uncertainty estimates. Hierarchical meta-learning therefore regulates the extent to which priors and evidence influence subsequent inference as a function of accumulated prediction history and inferred environmental structure.

Prediction error is defined in Equation 11, where $\kappa_{t}$ represents the expected uncertainty or informativeness of the environment. Since the posterior probability of an observation $\hat{P}(e_{t})$ is generated from beliefs updated by the log-likelihood ratio ${\mathrm{LLR}}_{e_{t}}$ , the statistical structure of evidence directly shapes higher-order reliability dynamics. Informative environments, characterized by consistently large likelihood ratios, progressively reduce estimated environmental uncertainty, whereas noisy environments with weak likelihood ratios maintain elevated uncertainty estimates.

This distinction yields qualitatively different learning regimes. Under low- $\kappa_{t}$ conditions, which correspond to highly informative environments with discriminant likelihood structures, even moderate prediction mismatches produce negative prediction errors. Therefore, reliability learning becomes increasingly selective; only highly predictive informational sources retain influence over time. Salient evidence initially amplifies evidence weighting because attentional salience increases the utility of incoming information (see Equation 8b). However, as repeated exposure stabilizes reliability estimates, inferential weighting shifts progressively toward accumulated predictive structure. Consequently, the effect of salience diminishes and may eventually reverse, despite initially strong evidence weighting.

In contrast, under high- $\kappa_{t}$ conditions, which correspond to noisy environments with weak likelihood ratios, prediction errors are comparatively more tolerant of mismatch. Reliability estimates accumulate more diffusely under these conditions, which preserves sensitivity to incoming evidence and sustains the impact of salient cues throughout the learning process. Salience continues to promote evidence weighting rather than being overridden by a stabilized inferential structure under these conditions.

The hierarchical interaction between environmental uncertainty, salience, and learning history emerges directly from the dynamics of reliability learning based on likelihood. Specifically, the framework predicts that, in highly informative environments characterized by discriminant likelihood structure, salience-driven amplification of evidence weighting should attenuate or reverse over time, but persist in noisy environments characterized by weak likelihood ratios. This uncertainty-by-salience-by-time-interaction is a key discriminative prediction of the hierarchical ABC framework because competing approaches generally predict monotonic modulation of evidence salience rather than dynamic reversal arising from higher-order uncertainty adaptation (see Section 4).

As an aside, the present framework also suggests a connection to cue competition phenomena in associative and contingency learning (Kopp and Reischies, 2000; Kopp and Wolff, 2000; Kopp et al., 2002; Kopp, 2007). Classical associative-learning models propose that cues compete for predictive strength as a function of prediction error and prior learning history (e.g., blocking effects; Rescorla and Wagner, 1972). Within the hierarchical ABC framework, analogous dynamics emerge from $\kappa_{t}$ -dependent reliability learning. Low- $\kappa_{t}$ environments characterized by stable and highly diagnostic likelihood structures promote selective reliability updating, such that informational sources with consistently strong predictive value increasingly dominate inferential weighting, whereas weaker or redundant cues progressively lose influence. Salient cues may initially capture inferential resources, but their long-run impact depends on their unique contribution to prediction error-reduction across learning history. By contrast, high- $\kappa_{t}$ environments maintain more diffuse reliability learning, reducing competitive exclusion among cues and preserving broader sensitivity to multiple informational sources. From this perspective, classical cue-competition effects in associative and human contingency learning may reflect the same higher-order uncertainty-dependent reliability dynamics that govern probabilistic inference more generally.

### 3.3 Prediction error-based learning of information reliability

The ABC framework suggests that, rather than making direct changes to the weighting parameter, the learning process operates on the inferred reliability of the underlying information sources (Equations 13, 14a, 14b, 15a, 15b). A simple, error-driven learning rule for reliability can be expressed as follows:


$$r_{i_{t+1}}=r_{i_{t}}+\eta \;\delta_{t}\;A_{i_{t}},\;i\in \{\text{prior},\text{evidence}\},$$ (13)

where for simplicity, we assume that the learning rate η is a constant, and $A_{i_{t}}$ is an attribution signal. A natural specification is η


$$A_{{\text{prior}}_{t}}=\gamma_{t}$$ (14a)

$$A_{{\text{evidence}}_{t}}=1-\gamma_{t}$$ (14b)

Substituting Equations 14a, 14b into Equation 13 yields


$$r_{{\text{prior}}_{t+1}}=r_{{\text{prior}}_{t}}+\eta \;\delta_{t}\;\gamma_{t}$$ (15a)

$$r_{{\text{evidence}}_{t+1}}=r_{{\text{evidence}}_{t}}+\eta \;\delta_{t}\;(1-\gamma_{t})$$ (15b)

**Figure 2:**
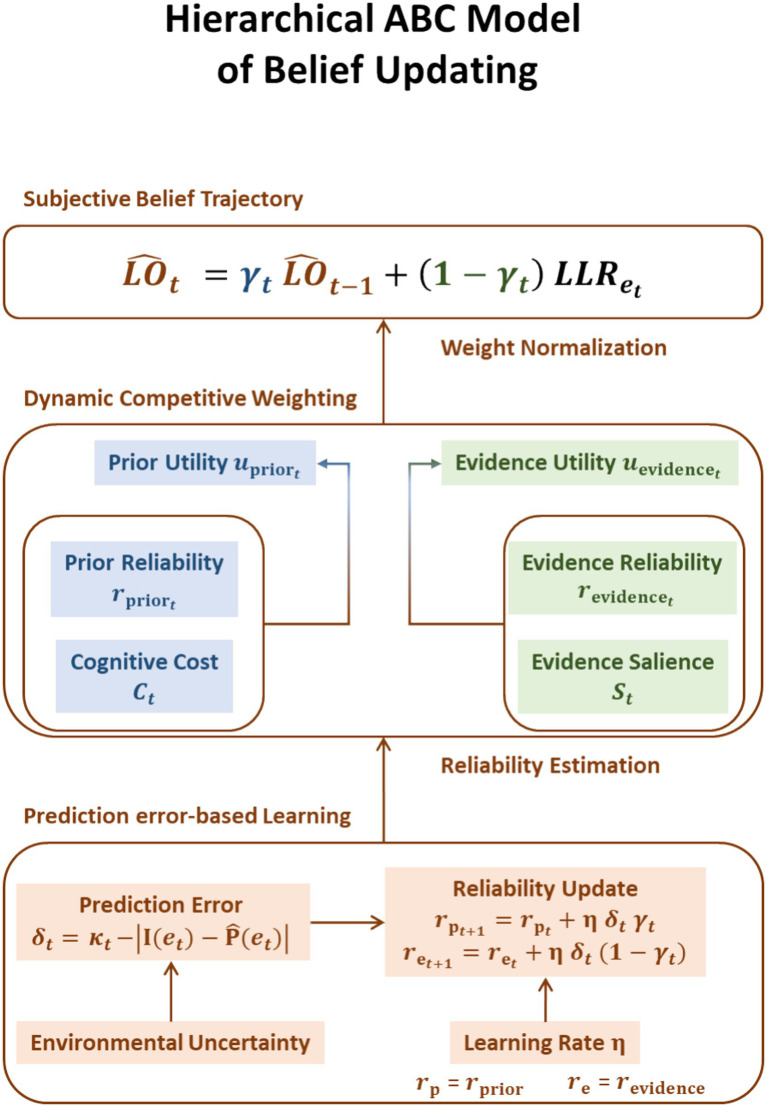
Recursive Bayesian inference across three hierarchical strata. The figure illustrates the hierarchical ABC framework, in which belief updating is formalized as a recursively weighted Bayesian process. At the first stratum, prior beliefs and incoming evidence are integrated in log-odds space through dynamically weighted updating. The weighting parameter $\gamma_{t}$ is determined by competitive valuation of prior- and evidence-based informational sources. Prior utility depends on estimated prior reliability and cognitive cost, whereas evidence utility depends on estimated evidence reliability and attentional salience. At the second stratum, prediction errors drive reliability learning by updating inferred trustworthiness of prior and evidence channels. At the third stratum, estimated environmental uncertainty $\kappa_{t}$ modulates the long-run dynamics of inferential weighting across learning history. Together, the framework links probabilistic inference, attentional salience, cognitive cost, reliability learning, and uncertainty adaptation within a unified hierarchical architecture.

As shown in Figure 2, inferential weighting arises from interactions across the three hierarchical strata of the ABC framework. Reliability estimates determine the utility of prior and evidence-based informational sources, which are normalized into the weighting parameter $\gamma_{t}$ , which governs belief updating. Prediction errors recursively update these reliability estimates. Meanwhile, higher-order estimates of environmental uncertainty $\kappa_{t}$ modulate their long-run dynamics across learning history. Thus, variability in probability weighting reflects context-sensitive and experience-dependent adaptation rather than fixed deviations from rationality.

Within the hierarchical ABC framework, prediction errors do not directly modify beliefs but instead update second-order reliability estimates associated with prior beliefs and incoming evidence. These learned reliability estimates dynamically regulate the weighting of informational sources during subsequent inference. Consequently, cognitive biases emerge as context-sensitive adaptations shaped by the statistical structure of the environment rather than as fixed deviations from rationality. Through repeated prediction error-based calibration, the system progressively adjusts inferential weighting to environmental regularities. From this perspective, expertise reflects refined reliability estimation acquired through extended exposure to informative feedback, yielding increasingly adaptive integration of informational sources under uncertainty.

ABC specifies a hierarchical computational architecture through which diverse psychological influences shape adaptive probabilistic inference. The hierarchical framework distinguishes three interacting computational strata. At the first level, beliefs are updated through the weighted integration of priors and evidence. The second level involves prediction errors driving learning about the reliability of informational sources. The third level involves the system adapting to the long-term uncertainty of the environment. The interaction among these strata generates inferential dynamics that cannot be reduced to any single component in isolation.

Importantly, the weighting parameter is not interpreted as a primitive cognitive resource or a single causal mechanism underlying all biases. Rather, it functions as a latent computational arbitration variable that governs the relative influence of priors and evidence during belief updating. This arbitration process may be influenced by multiple upstream factors, including attentional salience, memory accessibility and cognitive cost, learned reliability, and contextual uncertainty. These influences do not become part of the weighting parameter itself. Rather, they contribute to distinct utility and reliability estimates that determine inferential weighting together. Thus, ABC does not reduce all cognitive biases to a single mechanism. Rather, ABC specifies a common inferential architecture through which diverse cognitive influences generate systematic variation in probabilistic inference.

Compared to earlier formulations (Kopp, 2025), the current framework introduces several extensions. First, it establishes a clear hierarchical distinction between inferential updating, reliability learning, and uncertainty adaptation. Second, inferential weighting is formalized as emerging from competitive, utility-based arbitration between prior- and evidence-based informational sources, rather than from fixed or externally imposed gain parameters. Third, the current formulation emphasizes discriminability from competing frameworks by deriving qualitative dynamical predictions that distinguish ABC from static tempered Bayesian models and local precision-weighting accounts. These accounts are discussed in the subsequent section. Finally, the framework situates ABC more explicitly within broader contemporary discussions of ecological rationality, resource-rational cognition, predictive processing, and hierarchical inference. Thus, the goal is not merely to describe cognitive biases in Bayesian terms but also to specify a hierarchical computational architecture capable of generating distinctive adaptive dynamics across changing informational environments.

## 4 Adaptive Bayesian cognition across theoretical traditions in the field

The ABC framework is based on von Helmholtz's (1867) idea that perception is an unconscious process of making perceptual inferences. This process involves integrating prior knowledge and sensory evidence to determine the underlying causes of observed input. The ABC framework extends this inferential perspective by integrating insights from the heuristics-and-biases tradition, ecological rationality, Bayesian cognition, and resource-rational analysis. Rather than viewing cognitive biases as departures from probabilistic reasoning, the ABC framework conceptualizes them as systematic outcomes of adaptive Bayesian inference under resource constraints. Within this framework, biases such as representativeness and conservatism reflect different weighting regimes. Evidence-dominant updating increases the influence of likelihood information, while prior-dominant updating increases the influence of accumulated beliefs. Thus, cognitive biases emerge as lawful variations in probabilistic inference rather than failures of rational reasoning.

This reinterpretation aligns ABC closely with ecological-rationality approaches that emphasize the adaptive fit between cognition and environmental structure (Gigerenzer and Brighton, 2009; Brighton and Gigerenzer, 2015). However, ABC departs from traditional ecological-rationality accounts by embedding these adaptive patterns within a unified inferential architecture, rather than treating them as separate heuristic strategies. Environmental regularities modulate the allocation of inferential resources across informational sources, thereby altering the relative weighting of priors and evidence. Thus, adaptation is achieved through the continuous recalibration of inferential weighting rather than through the selection of separate algorithms.

The framework extends Bayesian approaches to cognition further by relaxing the assumption of normatively fixed integration. Bayesian models have demonstrated that human cognition often approximates probabilistic inference in domains including perception, causal learning, and inductive reasoning (Oaksford and Chater, 2007; Tenenbaum et al., 2011; Griffiths et al., 2024). Yet, many Bayesian formulations implicitly assume stable integration rules between priors and likelihoods. ABC, however, proposes that inferential weighting evolves dynamically as a function of attentional salience, cognitive cost, learned reliability, and environmental uncertainty. Thus, the framework preserves the probabilistic structure of inference while allowing lawful and systematic departures from normative Bayesian updating under constrained cognition.

In this regard, ABC can be viewed as an extension of the resource-rational approach (Lieder and Griffiths, 2020). Resource-rational approaches characterize cognition as optimization under limitations in time, memory, and computational capacity. ABC formalizes these constraints as a competitive inferential process in which priors and evidence both draw from the same limited pool of computational resources. Consequently, amplifying one informational source necessarily reduces the effective contribution of another. Unlike classical Bayesian models based on fixed additive accumulation, the ABC framework conceptualizes inference as adaptive arbitration between competing informational channels, whose relative influence is dynamically regulated across learning and uncertainty contexts.

Taken together, the ABC framework integrates three foundational insights: human judgment systematically deviates from normative benchmarks; nevertheless, these deviations may be adaptive given environmental structure; and cognition remains fundamentally probabilistic in form. Therefore, the central theoretical question shifts from whether human reasoning conforms to Bayesian principles to how cognitive constraints dynamically regulate the weighting of probabilistic information across contexts, timescales, and uncertainty regimes. These questions are addressed through the hierarchical extension of the ABC framework (see Section 3).

### 4.1 Related domains: attention, learning, and evidence accumulation

The hierarchical ABC framework has formal similarities with several influential models of attention, learning, and sequential inference, but differs in its treatment of adaptive inferential weighting. At the attentional level, the framework aligns with biased-competition models (Desimone and Duncan, 1995) in that it conceptualizes priors and incoming evidence as competing informational sources. Salient evidence biases processing toward likelihood-based updating, whereas highly accessible or strongly established priors bias processing toward accumulated internal representations. Thus, inferential weighting emerges from the competitive allocation of limited processing resources across informational channels.

At the learning level, the framework resembles associative learning models, in which prediction errors drive adaptive updating (Rescorla and Wagner, 1972). ABC generalizes this principle within a hierarchical probabilistic architecture by allowing prediction errors to regulate the inferred reliability of informational sources rather than merely associative strength. In this respect, the framework is conceptually related to attentional learning theories in which learning rates vary according to stimulus relevance or uncertainty (Mackintosh, 1975; Pearce and Hall, 1980). However, ABC embeds these dynamics within recursive Bayesian belief updating. This links attentional modulation, reliability learning, and probabilistic inference within a common computational structure.

The framework shares important properties with recursive Bayesian filtering and sequential accumulation models (Dayan and Abbott, 2001). As with standard filtering approaches, beliefs are updated sequentially over time by integrating prior expectations and incoming evidence. However, unlike classical Bayesian filters, ABC introduces adaptive inferential arbitration through the dynamic weighting of informational sources. Under prior-dominant regimes, belief trajectories become stable and resistant to change, resembling conservative or leaky accumulation dynamics (Usher and McClelland, 2001). Under evidence-dominant regimes, belief updating becomes increasingly sensitive to incoming observations, facilitating rapid revision. Thus, sequential accumulation emerges not from fixed integration rules but from the adaptive regulation of inferential weighting across learning contexts.

At the algorithmic level, ABC resembles sequential sampling frameworks, such as the Sequential Probability Ratio Test (Wald, 1947) and the Drift–Diffusion Model (Ratcliff and McKoon, 2008). Both of these models characterize decisions as arising through the gradual accumulation of evidence over time. However, whereas standard accumulation models typically assume stationary accumulation dynamics, ABC proposes that the effective contribution of priors and evidence evolves as a function of learned reliability, salience, and environmental uncertainty. Thus, the framework extends conventional accumulation approaches (Bogacz et al., 2006; Brown and Heathcote, 2008) by embedding evidence accumulation within a hierarchical system of adaptive probabilistic weighting.

### 4.2 Related neurocognitive models

In contemporary neuroscience, these ideas have been formalized by the Bayesian brain hypothesis. According to this hypothesis, the nervous system performs probabilistic computations to manage uncertainty in neural coding (Knill and Pouget, 2004). This perspective culminates in predictive processing and free-energy accounts, which conceptualize biological systems as inference engines that minimize discrepancies between internally generated predictions and sensory input by minimizing prediction errors (Friston, 2010). Prediction errors thereby constitute the primary signal for updating internal models.

The hierarchical ABC framework aligns with these approaches, as well as with neurocognitive theories that interpret cognitive control as the dynamic allocation of processing resources based on the expected utility and reliability of information (Koechlin and Summerfield, 2007; Shenhav et al., 2013). Within ABC, reliability estimates regulate the relative weighting of priors and evidence, thereby integrating reliability learning, resource allocation, and probabilistic belief updating within a common hierarchical architecture.

ABC parallels precision-weighting accounts in predictive processing and free-energy models, in which the impact of prediction errors is scaled by estimated precision (Friston, 2010; Clark, 2013; Parr et al., 2022; Butz et al., 2025). However, an important difference concerns the structure of inferential modulation. In predictive processing formulations, precision primarily regulates the gain of likelihood-derived prediction errors while preserving the structural role of priors within the generative model. In contrast, ABC conceptualizes priors and evidence as competing informational sources whose relative influence is dynamically reallocated across learning contexts through adaptive inferential weighting. Reliability estimates thus serve not only as precision-like quantities that regulate update strength but also as determinants of source dominance within inference itself. In this respect, the framework integrates principles from Bayesian cognition, predictive coding, ecological rationality, and resource-rational analysis within a common hierarchical architecture (Knill and Pouget, 2004; Gigerenzer and Brighton, 2009; Lieder and Griffiths, 2020).

Despite this distinction, both frameworks converge on a common computational principle: context-sensitive weighting of information under uncertainty. Predictive processing implements this principle through the propagation of prediction errors weighted by precision, whereas hierarchical ABC implements it through the arbitration of priors and evidence based on reliability. In both cases, cognitive biases emerge not as violations of probabilistic reasoning but as adaptive consequences of dynamically weighted inference under uncertainty (Hohwy, 2013).

### 4.3 Allied disciplines: behavioral economics, engineering, and artificial intelligence

In hierarchical ABC, priors and evidence are treated as competing informational streams that are integrated under resource constraints. Inferential weighting is dynamically regulated by learned reliability. Although belief integration is locally linear in log-odds space, the nonlinear mapping between log-odds and probability space implies that adaptive changes in inferential weighting produce nonlinear, often inverse, S-shaped behavioral probability weighting (Kopp, 2025). Importantly, these distortions arise from reliability-modulated inference rather than primitive transformations of probability itself.

Importantly, the hierarchical ABC model does not treat inferential weighting as a primitive, free parameter, nor as a direct transformation of objective probabilities into distorted, subjective decision weights. This is in contrast to classical probability-weighting formulations (Goldstein and Einhorn, 1987; Tversky and Kahneman, 1992; Gonzalez and Wu, 1999; Wakker, 2010; Zhang and Maloney, 2012). Rather, weighting emerges from the interactions of belief updating, reliability learning, and uncertainty adaptation within a hierarchical structure. Thus, cognitive biases arise not because probabilistic reasoning is abandoned but because probabilistic inference itself is adaptively shaped by resource limitations, learning dynamics, and environmental structure.

This framework bears formal similarities to recursive Bayesian filtering and weighted Bayesian inference. In Kalman filtering, the Kalman gain determines the relative contribution of prior estimates and observations as a function of uncertainty (Kalman, 1960). ABC shares this principle of reliability-weighted updating, but it extends beyond fixed Gaussian assumptions by treating reliability as a dynamically learned, context-sensitive control variable. Similarly, tempered Bayesian approaches modulate the influence of likelihood information through temperature parameters (Alquier, 2020, 2024; Bissiri et al., 2016). However, ABC implements a formally related yet conceptually distinct mechanism. Inferential weighting emerges endogenously through adaptive competition between priors and evidence, rather than through externally specified likelihood tempering alone.

The distinctive contribution of the hierarchical ABC framework is not the introduction of entirely novel computational primitives, but rather the coupling of inferential updating, reliability learning, and uncertainty adaptation within a three-strata architecture. This organization generates an empirical prediction that is absent from most competing frameworks: the effect of salience on evidence weighting should evolve dynamically as a function of both environmental uncertainty and accumulated learning history. In highly informative environments, salience initially amplifies evidence weighting, but later diminishes or reverses its influence as stable predictive structures increasingly dominate inference. In noisy environments, however, salience effects are predicted to persist or strengthen over time. This interaction between salience, uncertainty, and time is a key discriminative prediction of the ABC framework because competing tempered Bayesian, Kalman filtering, and predictive processing accounts typically predict monotonic, stationary weighting dynamics, gain modulation, or local precision adaptation. These accounts do not generally predict the selective crossover behavior that emerges from hierarchical uncertainty learning.

## 5 Adaptive Bayesian cognition across individuals and ecological contexts

The hierarchical ABC framework explains systematic variation in probabilistic inference as a consequence of adaptive weighting between priors and evidence under resource constraints. Inferential updating is governed by a dynamically calibrated weighting process influenced by higher-order estimates of cognitive cost, attentional salience, informational reliability, and environmental uncertainty. Thus, stable individual differences in reasoning emerge from differences in how cognitive resources are allocated across competing informational sources.

At the first level of the hierarchy, the cognitive system integrates prior beliefs with incoming evidence to generate updated judgments. At higher levels, the system estimates the reliability of informational sources and tracks their predictive success across contexts and time. These meta-level processes dynamically calibrate inferential weighting through prediction error-based learning. Consequently, probabilistic inference is adaptively tuned to the statistical structure of the environment rather than fixed.

This framework naturally explains variability in reasoning performance. Integrating prior beliefs with diagnostic evidence places substantial demands on working memory and attentional control because multiple informational streams must be maintained simultaneously. Individuals with greater cognitive resources are more likely to approximate balanced Bayesian integration. In contrast, constrained processing favors extreme weighting regimes dominated by either priors or salient evidence. Expertise emerges when repeated exposure and reliable feedback stabilize higher-order estimates of informational reliability, producing increasingly calibrated inferential weighting over time.

The same hierarchical mechanism provides a computational account of psychopathology. According to predictive-processing theories, disturbances in precision assignment and prediction error-signaling lead to atypical belief formation (Corlett et al., 2010; Friston, 2010; Adams et al., 2013; Stephan and Mathys, 2014). ABC formalizes this proposal as distorted reliability learning within a unified inferential architecture. Overestimating prior reliability promotes rigid, prior-dominant belief states that are resistant to disconfirmatory evidence (Fletcher and Frith, 2009). On the other hand, excessive weighting of sensory prediction errors produces unstable, evidence-dominant inference that is overly sensitive to momentary input (Lawson et al., 2014). Thus, psychopathological reasoning is conceptualized as an extreme operating regime arising from the maladaptive calibration of reliability estimates (Hohwy, 2013), rather than as a distinct cognitive system.

Initial empirical support for the ABC account comes from probabilistic inference tasks, such as urn paradigms, in which observers integrate base-rate information with one-step (Kopp, 2025) or sequential evidence (Boos et al., 2016). Hierarchical modeling revealed substantial heterogeneity in inferential weighting across individuals and demonstrate that hierarchical Bayesian approaches consistently outperform models that assume homogeneous inferential parameters (Boos et al., 2016). In addition, Kopp and Wolff (2000) describe a prediction error-based mechanism in human contingency learning. In this paradigm, cue validity is updated through trial-by-trial error correction, resulting in competition among associative cues. Furthermore, Kopp and Reischies (2000), Kopp et al. (2002), and Kopp (2007) suggest that cognition within the schizophrenia spectrum is characterized by an inability to use prediction error signals to update the estimated reliability of competing associative cues, which depends on the maintenance of information in working memory. Consequently, cue validity is not revised adaptively over time, resulting in cue competition failing to evolve. This is consistent with a disruption to reliability learning in situations where cues compete to be associated with anticipated events.

Together, these frameworks provide a unified account of individual differences, expertise, and psychopathology based on hierarchical reliability learning. Apparent deviations from normative Bayesian reasoning are interpreted as adaptive - and sometimes maladaptive - consequences of resource-constrained probabilistic inference, rather than as failures of rationality.

The framework also has important implications for real-world decision-making. In domains such as medical diagnosis and financial forecasting, errors often stem from the mis-weighting of priors and salient evidence rather than an absence of normative knowledge. Thus, ABC suggests that effective decision-support systems should focus on improving the calibration of inferential weighting itself rather than merely presenting Bayesian solutions (Phillips-Wren et al., 2022). Because the ABC framework distinguishes between first-order inference and higher-order reliability learning, interventions can target either level by enhancing the representation of probabilistic information, providing feedback about predictive validity, or signaling the structure of uncertainty dynamically.

Similarly, this perspective reframes training as the calibration of meta-learning processes rather than the memorization of normative rules. Through repeated exposure to environments that differ in the reliability of priors and evidence, combined with feedback on predictive performance, learners can acquire adaptive weighting strategies. Since inferential weighting can be estimated at the individual level using hierarchical Bayesian methods, decision support and training systems can be personalized to detect and correct systematic weighting distortions.

In artificial intelligence, these ideas naturally connect to tempered Bayesian inference, in which the impact of evidence is controlled through temperature parameters specified externally (Alquier, 2020). ABC extends this logic by treating inferential weighting as learned dynamically through hierarchical reliability adaptation rather than being fixed *a priori*. This distinction suggests a path toward AI systems that can continuously recalibrate the balance between priors and evidence as environmental structures change. More broadly, the framework provides a common computational perspective that links cognitive psychology, neuroscience, clinical science, and adaptive artificial intelligence through the principle of hierarchical, reliability-weighted, probabilistic inference.

## 6 Limitations and future directions

The ABC framework provides a hierarchical account of probabilistic inference, in which the updating of beliefs emerges from the dynamic interplay of prior beliefs and evidence, subject to cognitive and environmental constraints. Several components of the framework are already well defined. Notably, the formulation of belief updating in log-odds space offers a precise computational description of how priors and likelihood information are integrated. Meanwhile, the hierarchical extension specifies how inferential weighting is influenced by higher-order reliability learning and uncertainty adaptation. Consequently, the framework generates clear qualitative predictions regarding the impact of salience, cognitive load and environmental structure on probabilistic reasoning.

ABC does not assume that subjective beliefs initially correspond to an ideally calibrated Bayesian observer. Rather, deviations from normative Bayesian inference are expected because posterior beliefs arise from the integration of prior expectations and incoming evidence, with their relative weighting varying across individuals and contexts. Accordingly, Bayesian inference serves as a computational reference model, whereas ABC formalizes the adaptive mechanisms through which the weighting of priors and evidence can produce variability in belief updating.

At the same time, important aspects of the framework require further theoretical and empirical development. Most critically, the determinants of adaptive inferential weighting are only partially specified. Although the framework acknowledges attentional salience, cognitive costs, learned reliability, and environmental uncertainty as important factors that influence inference, the precise computational interactions among these factors remain to be determined. The current formulation should therefore be regarded as an initial approximation of a broader, more comprehensive theory of adaptive probabilistic integration.

A particularly important unresolved issue concerns temporal dynamics. Many ecologically valid inference problems involve the accumulation of sequential evidence, requiring the continuous re-evaluation of the reliability of priors and evidence. The hierarchical ABC framework proposes that inferential weighting evolves through prediction error-based meta-learning processes that track the relative predictive success of competing informational sources over time. This proposal is consistent with hierarchical models of learning under uncertainty within the predictive processing and reinforcement learning traditions (Friston, 2010; Dolan and Dayan, 2013). However, direct empirical evidence for such adaptive reliability learning remains limited. To establish these dynamics, paradigms are needed that can manipulate the reliability of priors and evidence independently while tracking inferential weighting trial by trial across changing environments.

The present formulation of ABC should not be interpreted as proposing that the brain explicitly computes or continuously optimizes all variables specified by the framework. Instead, ABC distinguishes between latent descriptors of information-processing constraints and inferential quantities that may be subject to adaptive estimation. Cognitive cost and salience are not assumed to be directly computed by the brain; rather, they characterize emergent properties of processing. Cognitive cost reflects the intrinsic constraints associated with maintaining and updating prior representations, whereas salience captures the attentional relevance of incoming sensory information. By contrast, reliability and uncertainty represent inferential quantities that can be estimated, as adaptive belief updating requires assessing the relative contributions of prior knowledge and new evidence. Accordingly, ABC does not posit an additional resource-demanding control algorithm that regulates inference. Rather, it provides a computational-level account in which some variables describe the consequences of biased and constrained information processing, while others formalize the estimates required to adaptively weight information.

The framework also has important limitations in terms of its scope. Firstly, ABC is not intended to be a comprehensive theory of the processes underlying attention, memory, perception and motivation. Instead, it specifies a hierarchical inferential architecture through which these factors work together to regulate probabilistic updating. Secondly, the framework does not claim that all cognitive biases arise from a single primitive mechanism. Similar inferential weighting patterns may emerge from multiple upstream causes, including additional attentional, mnemonic, motivational, or environmental factors. Thirdly, this article primarily advances a theoretical and computational framework, rather than a comprehensive empirical model that can be applied to all domains of judgment and decision-making. Systematic quantitative comparisons with competing models therefore remain an important area for future research.

The present framework is intended primarily as a computational theory rather than a fully specified process model. Its aim is to define the hierarchical inferential architecture underlying adaptive Bayesian cognition while leaving the algorithmic implementation of upstream constructs, such as attentional salience and cognitive cost, open to future empirical investigation. Accordingly, these variables are treated as abstract computational quantities whose psychological and neural implementations may vary across cognitive domains. In contrast, environmental uncertainty is formalized as a higher-order state variable governing long-term reliability learning. We define environmental uncertainty, *κ*_t_, as the expected informativeness of the environment, which may be approximated by the expected absolute log-likelihood ratio of incoming evidence, $\kappa_{t}$ ≈ (| ${\mathrm{LLR}}_{e_{t}}$ |). Within the hierarchical architecture, $\kappa_{t}$ regulates the stability of reliability learning across extended experience while remaining distinct from trial-level belief updating.

Despite these limitations, the framework yields several empirically discriminative predictions. For example, increased salience or perceived reliability of evidence should bias inference toward evidence-dominant updating, whereas explicit representation of base rates should strengthen prior reliance. High cognitive load should promote selective weighting and increased single-source dominance. Furthermore, the hierarchical extension predicts that salience effects should evolve dynamically in response to environmental uncertainty and learning history. In highly informative environments, salience is initially predicted to enhance evidence weighting, but this effect is expected to diminish or reverse as stable predictive structure becomes increasingly dominant. In contrast, under noisy environments, salience effects should persist or strengthen over time. This predicted interaction between salience, uncertainty and time constitutes a key discriminative signature of the framework relative to tempered Bayesian, predictive-processing and resource-rational alternatives.

Further empirical work is needed to determine the extent to which ABC generates predictions that can be distinguished from those of related hierarchical Bayesian and predictive processing accounts. We have clarified the framework’s discriminative predictions and identified their empirical evaluation as a key direction for future research. While the current framework yields several predictions that diverge from those of related approaches, these distinctions should be considered theoretical hypotheses rather than established empirical facts. Accordingly, the current work does not claim that the proposed learning dynamics are uniquely generated by ABC. Rather, it identifies candidate discriminative predictions that can be evaluated in future experiments designed to directly compare competing computational frameworks. An important objective for future empirical research is establishing the specificity of these predictions and determining which aspects of adaptive inference are unique to ABC versus those shared with related models.

We acknowledge that the current formulation does not yet constitute a fully specified computational model with a fixed parameter set and an explicit implementation algorithm. This is an intentional characteristic of ABC at its present stage of development. Rather than serving as a complete process-level model, the framework is primarily conceptual, aiming to define the computational principles and organizational structure that underlie adaptive inference. The proposed latent variables—including uncertainty, reliability, salience, and cognitive cost—should therefore be understood as theoretical constructs that formalize the mechanisms through which adaptive weighting of information may be organized in response to changing environmental demands. Their role is to establish testable relationships and generate qualitative predictions, while leaving their precise parameterization and neural implementation open for future empirical investigation and computational refinement.

The framework also naturally extends to psychopathology. According to predictive-processing theories, psychopathological belief formation is linked to disturbances in precision assignment and prediction error-signaling (Fletcher and Frith, 2009; Friston, 2010; Adams et al., 2013; Stephan and Mathys, 2014). ABC reformulates these disturbances as maladaptive reliability learning within a unified inferential architecture. Excessive reliance on priors can lead to rigid, delusion-like interpretations that are resistant to disconfirmatory evidence. Conversely, excessive weighting of sensory evidence can generate unstable and overly context-sensitive belief states (Lawson et al., 2014). Consequently, psychopathology is not conceptualized as a qualitatively distinct reasoning system, but rather as an extreme operating regime that arises from the distortion of higher-order calibration of informational reliability (Hohwy, 2013).

The framework initially received empirical support from probabilistic inference paradigms, such as urn tasks involving the sequential integration of base rates and sampled evidence (Boos et al., 2016; Kolossa et al., 2015). Neurophysiological evidence suggests that distinct components of the P300 complex track complementary aspects of probabilistic updating and prediction error-processing (Friston, 2005; Kopp, 2008; Kopp et al., 2016; Seer et al., 2016; Visalli et al., 2021, 2023). In particular, Kolossa et al. (2015) reported distinct neural signatures associated with updating beliefs, making predictive adjustments and trial-wise prediction errors during probabilistic inference. However, these findings were obtained under stationary inferential assumptions and therefore do not directly address the temporal dynamics of adaptive reliability learning proposed by the hierarchical extension.

At this stage, ABC is intended as a computational framework rather than a fully parameterized model with a universal set of fixed values applicable across domains. Its primary objective is to specify the computational organization of adaptive inference and to generate testable predictions concerning the interactions among salience, cognitive cost, reliability, uncertainty, and belief updating. Empirical validation should therefore initially focus on paradigms that provide direct access to the inferential quantities formalized by the framework, such as trial-by-trial estimates of posterior probabilities in Bayesian urn-inference tasks. Such paradigms provide controlled benchmarks for evaluating the proposed computational principles and their neural correlates, including trial-by-trial variability in the P300 as a potential marker of adaptive inferential weighting. Extending ABC to more complex, real-world judgments will require domain-specific scaling and parameterization, as the structure, dynamics, and reliability of inferential environments are expected to shape the relative contributions of different computational variables.

The three-strata architecture of ABC generalizes these findings by proposing that inferential weighting evolves through the interaction of three processes: first-order belief updating, second-order reliability learning, and higher-order uncertainty adaptation. This formulation yields concrete neurocomputational predictions. For instance, greater prior dominance attenuates evidence-driven prediction error-signals, while evidence-dominant regimes amplify updating-related neural responses. More broadly, adaptive changes in inferential weighting are expected to systematically modulate neural markers of prediction, uncertainty, and belief revision across contexts and learning phases.

Together, these findings provide a unifying computational perspective linking probabilistic reasoning, learning, psychopathology, and neural implementation through hierarchical, reliability-weighted inference. Advancing this program will require coordinated behavioral, computational, and neurophysiological research capable of testing how inferential weighting evolves dynamically across uncertainty regimes and learning histories.

## 7 Conclusion and implications

The hierarchical ABC framework reconceptualizes human probabilistic reasoning as a dynamically regulated process involving reliability-weighted inferences across levels of belief updating, reliability learning, and uncertainty adaptation. Within this framework, cognitive biases are not merely violations of rational principles. Instead, they emerge as lawful consequences of adaptive inferential weighting under constraints of cognitive capacity, attentional selection, and environmental uncertainty. Prediction error-based learning continuously recalibrates the relative influence of priors and evidence, producing context-sensitive shifts in probabilistic reasoning over time and in different environments.

This perspective unifies several major traditions within a common computational framework. It preserves the empirical phenomena emphasized by the heuristics-and-biases tradition, retains the probabilistic foundations of Bayesian cognition, aligns with ecological and resource-rational approaches that emphasize adaptive behavior under constraints, and converges with predictive-processing accounts of reliability- and precision-weighted inference. Simultaneously, the hierarchical extension specifies how these processes dynamically interact through the higher-order learning of informational reliability and environmental uncertainty.

Within this framework, systematic variation in inferential weighting gives rise to distinct reasoning regimes associated with phenomena such as conservatism, base-rate neglect, anchoring, and jumping to conclusions. Thus, these effects are not interpreted as isolated heuristics, but rather as emergent properties of a unified inferential architecture that operates under different informational and ecological conditions.

The implications of this framework extend across levels of analysis. In cognitive psychology, for example, it provides a quantitative account of graded variation in belief updating. In neuroscience, it links probabilistic inference to mechanisms of prediction error-processing, precision control, and attentional modulation. In psychopathology, it allows us to understand maladaptive belief formation as distorted higher-order calibration of informational reliability. In the field of artificial intelligence, the ABC framework employs a method of tempered Bayesian inference that is dynamically adaptive. This method treats inferential weighting as an endogenous property that is learned by the system over time.

Together, the hierarchical ABC framework presents a broader theoretical perspective in which human cognition is fundamentally probabilistic, yet adaptively influenced by resource limitations, attentional factors, learning history, and environmental structure. Thus, cognitive biases are best understood as systematic outcomes of hierarchical inference under uncertainty rather than as irrational anomalies.

## Data Availability

The original contributions presented in the study are included in the article/supplementary material, further inquiries can be directed to the corresponding author.

## References

[ref1] Adams R. Stephan K. Brown H. Frith C. Friston K. (2013). The computational anatomy of psychosis. Front. Psych. 4:47. doi: 10.3389/fpsyt.2013.00047, 23750138 PMC3667557

[ref2] Alquier P. (2020). Approximate Bayesian inference. Entropy 22:1272. doi: 10.3390/e22111272, 33287041 PMC7711853

[ref3] Alquier P. (2024). User-friendly introduction to PAC-Bayes bounds. Found. Trends Mach. Learn. 17, 174–303. doi: 10.1561/2200000100

[ref4] Anderson C. (2007). “Belief perseverance,” in Encyclopedia of Social Psychology, eds. Baumeister R. Vohs K. (Thousand Oaks, CA: Sage), 109–110.

[ref5] Bar-Hillel M. (1980). The base-rate fallacy in probability judgments. Acta Psychol. 44, 211–233. doi: 10.1016/0001-6918(80)90046-3

[ref6] Baron J. (2023). Thinking and Deciding. 5th Edn Cambridge, UK: Cambridge University Press.

[ref7] Bissiri P. Holmes C. Walker S. (2016). A general framework for updating belief distributions. J. R. Stat. Soc. Ser. B Stat. Methodol. 78, 1103–1130. doi: 10.1111/rssb.12158, 27840585 PMC5082587

[ref8] Bogacz R. Brown E. Moehlis J. Holmes P. Cohen J. (2006). The physics of optimal decision making: a formal analysis of models of performance in two-alternative forced-choice tasks. Psychol. Rev. 113, 700–765. doi: 10.1037/0033-295X.113.4.700, 17014301

[ref9] Boos M. Seer C. Lange F. Kopp B. (2016). Probabilistic inference: task dependency and individual differences of probability weighting revealed by hierarchical Bayesian modeling. Front. Psychol. 7:755. doi: 10.3389/fpsyg.2016.00755, 27303323 PMC4882416

[ref10] Brighton H. Gigerenzer G. (2015). The bias bias. J. Bus. Res. 68, 1772–1784. doi: 10.1016/j.jbusres.2015.01.061

[ref11] Brown S. Heathcote A. (2008). The simplest complete model of choice response time: Linear ballistic accumulation. Cogn. Psychol. 57, 153–178. doi: 10.1016/j.cogpsych.2007.12.002, 18243170

[ref12] Brunswik E. (1943). Organismic achievement and environmental probability. Psychol. Rev. 50, 255–272. doi: 10.1037/h0060889

[ref13] Brunswik E. (1952). The conceptual framework of psychology. Chicago, IL: University of Chicago Press.

[ref14] Butz M. Mittenbühler M. Schwöbel S. Achimova A. Gumbsch C. Otte S. . (2025). Contextualizing predictive minds. Neurosci. Biobehav. Rev. 168:105948. doi: 10.1016/j.neubiorev.2024.105948, 39580009

[ref15] Clark A. (2013). Whatever next? Predictive brains, situated agents, and the future of cognitive science. Behav. Brain Sci. 36, 181–204. doi: 10.1017/S0140525X12000477, 23663408

[ref16] Corlett P. Taylor J. Wang X. Fletcher P. Krystal J. (2010). Toward a neurobiology of delusions. Prog. Neurobiol. 92, 345–369. doi: 10.1016/j.pneurobio.2010.06.007, 20558235 PMC3676875

[ref17] Dayan P. Abbott L. (2001). Theoretical Neuroscience: Computational and Mathematical Modeling of Neural Systems. Cambridge, MA: MIT Press.

[ref18] Desimone R. Duncan J. (1995). Neural mechanisms of selective visual attention. Annu. Rev. Neurosci. 18, 193–222. doi: 10.1146/annurev.ne.18.030195.001205, 7605061

[ref19] Dolan R. Dayan P. (2013). Goals and habits in the brain. Neuron 80, 312–325. doi: 10.1016/j.neuron.2013.09.007, 24139036 PMC3807793

[ref20] Fennell J. Baddeley R. (2012). Uncertainty plus prior equals rational bias: an intuitive Bayesian probability weighting function. Psychol. Rev. 119, 878–887. doi: 10.1037/a0029346, 22800412

[ref21] Fiedler K. Juslin P. (2006). Information sampling and adaptive cognition. Cambridge, UK: Cambridge University Press.

[ref22] Fletcher P. Frith C. (2009). Perceiving is believing: a Bayesian approach to explaining the positive symptoms of schizophrenia. Nat. Rev. Neurosci. 10, 48–58. doi: 10.1038/nrn2536, 19050712

[ref23] Friston K. (2005). A theory of cortical responses. Philos. Trans. R. Soc. B Biol. Sci. 360, 815–836. doi: 10.1098/rstb.2005.1622, 15937014 PMC1569488

[ref24] Friston K. (2010). The free-energy principle: a unified brain theory? Nat. Rev. Neurosci. 11, 127–138. doi: 10.1038/nrn2787, 20068583

[ref25] Garety P. Freeman D. Jolley S. Ross K. Waller H. Dunn G. (2011). Jumping to conclusions: the psychology of delusional reasoning. Adv. Psychiatr. Treat. 17, 332–339. doi: 10.1192/apt.bp.109.007104

[ref26] Gershman S. Horvitz E. Tenenbaum J. (2015). Computational rationality: A converging paradigm for intelligence in brains, minds, and machines. Science 349, 273–278. doi: 10.1126/science.aac6076, 26185246

[ref27] Gigerenzer G. Brighton H. (2009). Homo heuristicus: why biased minds make better inferences. Top. Cogn. Sci. 1, 107–143. doi: 10.1111/j.1756-8765.2008.01006.x, 25164802

[ref28] Gilovich T. Griffin D. Kahneman D. (2002). Heuristics and biases: The psychology of intuitive judgment. Cambridge, UK: Cambridge University Press.

[ref29] Goldstein W. Einhorn H. (1987). Expression theory and the preference reversal phenomena. Psychol. Rev. 94, 236–254. doi: 10.1037//0033-295x.94.2.236

[ref30] Gonzalez R. Wu G. (1999). On the shape of the probability weighting function. Cogn. Psychol. 38, 129–166. doi: 10.1006/cogp.1998.0710, 10090801

[ref31] Griffiths T. Chater N. Tenenbaum J. (Eds.). (2024). Bayesian Models of Cognition: Reverse Engineering the Mind. Cambridge, MA: MIT Press.

[ref33] Hohwy J. (2013). The Predictive Mind. Oxford, UK: Oxford University Press.

[ref34] Jaynes E. (2003). Probability Theory: The Logic of Science. Cambridge, UK: Cambridge University Press.

[ref35] Juslin P. (2015). Controlled information integration and Bayesian inference. Front. Psychol. 6:70. doi: 10.3389/fpsyg.2015.00070, 25698998 PMC4316708

[ref36] Juslin P. Karlsson L. Olsson H. (2008). Information integration in multiple cue judgment: a division of labor hypothesis. Cognition 106, 259–298. doi: 10.1016/j.cognition.2007.02.003, 17376423

[ref37] Kahneman D. (2003). Maps of bounded rationality: psychology for behavioral economics. Am. Econ. Rev. 93, 1449–1475. doi: 10.1257/000282803322655392

[ref38] eds. Kahneman D. Slovic P. Tversky A. (1982). Judgment Under Uncertainty: Heuristics and Biases. Cambridge, MA: Cambridge University Press. 10.1126/science.185.4157.1124 17835457

[ref39] Kahneman D. Tversky A. (1972). Subjective probability: a judgment of representativeness. Cogn. Psychol. 3, 430–454. doi: 10.1016/0010-0285(72)90016-3

[ref40] Kahneman D. Tversky A. (1973). On the psychology of prediction. Psychol. Rev. 80, 237–251. doi: 10.1037/h0034747

[ref41] Kalman R. (1960). A new approach to linear filtering and prediction problems. J. Basic Eng. 82, 35–45. doi: 10.1115/1.3662552

[ref42] Klayman J. (1995). Varieties of confirmation bias. Psychol. Learn. Motiv. 32, 385–418. doi: 10.1016/s0079-7421(08)60315-1

[ref43] Knill D. Pouget A. (2004). The Bayesian brain: the role of uncertainty in neural coding and computation. Trends Neurosci. 27, 712–719. doi: 10.1016/j.tins.2004.10.007, 15541511

[ref44] Koechlin E. Summerfield C. (2007). An information theoretical approach to prefrontal executive function. Trends Cogn. Sci. 11, 229–235. doi: 10.1016/j.tics.2007.04.005, 17475536

[ref46] Kolossa A. Kopp B. Fingscheidt T. (2015). A computational analysis of the neural bases of Bayesian inference. NeuroImage 106, 222–237. doi: 10.1016/j.neuroimage.2014.11.007, 25462794

[ref47] Kopp B. (2007). Mnemonic intrusions into working memory in psychometrically identified schizotypal individuals. J. Behav. Ther. Exp. Psychiatry 38, 56–74. doi: 10.1016/j.jbtep.2006.05.001, 16828703

[ref48] Kopp B. (2008). “The P300 component of the event-related brain potential and Bayes’ theorem,” in Cognitive Sciences at the Leading Edge, ed. Sun M. K. (New York: Nova Science Publishers), 87–96.

[ref49] Kopp B. (2025). Cognitive biases as Bayesian probability weighting in context. Front. Psychol. 16:1572168. doi: 10.3389/fpsyg.2025.1572168, 40842605 PMC12366471

[ref50] Kopp B. Reischies F. (2000). Experimental evidence for distorted associative memory in chronic schizophrenic patients. Cogn. Neuropsychiatry 5, 241–254. doi: 10.1080/13546800050199702

[ref51] Kopp B. Seer C. Lange F. Kluytmans A. Kolossa A. Fingscheidt T. . (2016). P300 amplitude variations, prior probabilities and likelihoods: A Bayesian ERP study. Cogn. Affect. Behav. Neurosci. 16, 911–928. doi: 10.3758/s13415-016-0442-3, 27406085

[ref52] Kopp B. Wolff M. (2000). Brain mechanisms of selective learning: event-related potentials provide evidence for error-driven learning in humans. Biol. Psychol. 51, 223–246. doi: 10.1016/s0301-0511(99)00039-3 10686367

[ref53] Kopp B. Wolff M. Hruska C. Reischies F. (2002). Brain mechanisms of visual encoding and working memory in psychometrically identified schizotypal individuals and after acute administration of haloperidol. Psychophysiology 39, 459–472. doi: 10.1111/1469-8986.3940459, 12212638

[ref54] Lawson R. Rees G. Friston K. (2014). An aberrant precision account of autism. Front. Hum. Neurosci. 8:302. doi: 10.3389/fnhum.2014.00302, 24860482 PMC4030191

[ref55] Lieder F. Griffiths T. (2020). Resource-rational analysis: understanding human cognition as the optimal use of limited computational resources. Behav. Brain Sci. 43, 1–60. doi: 10.1017/S0140525X1900061X, 30714890

[ref56] Lieder F. Griffiths T. Huys Q. Goodman N. (2018). The anchoring bias reflects rational use of cognitive resources. Psychon. Bull. Rev. 25, 322–349. doi: 10.3758/s13423-017-1286-8, 28484952

[ref57] Lindley D. (1985). Making decisions. Hoboken, NJ: Wiley.

[ref58] Mackintosh N. (1975). A theory of attention: variations in the associability of stimuli with reinforcement. Psychol. Rev. 82, 276–298. doi: 10.1037/h0076778

[ref59] Oaksford M. Chater N. (2007). Bayesian Rationality: The Probabilistic Approach to Human Reasoning. Oxford, UK: Oxford University Press. 10.1017/S0140525X09000284 19210833

[ref60] Parr T. Pezzulo G. Friston K. (2022). Active Inference: The Free energy Principle in Mind, Brain, and Behavior. Cambridge, MA: MIT Press.

[ref61] Pearce J. Hall G. (1980). A model for Pavlovian learning: variations in the effectiveness of conditioned but not of unconditioned stimuli. Psychol. Rev. 87, 532–552. doi: 10.1037/0033-295x.87.6.532, 7443916

[ref62] Peterson C. Beach L. (1967). Man as an intuitive statistician. Psychol. Bull. 68, 29–46. doi: 10.1037/h0024722, 6046307

[ref63] Phillips L. Edwards W. (1966). Conservatism in a simple probability inference task. J. Exp. Psychol. 72, 346–354. doi: 10.1037/h0023653, 5968681

[ref64] Phillips-Wren G. Daly M. Burstein F. (2022). Support for cognition in decision support systems: an exploratory historical review. J. Decis. Syst. 31, 18–30. doi: 10.1080/12460125.2022.2070946

[ref65] Pilgrim C. Sanborn A. Malthouse E. Hills T. (2024). Confirmation bias emerges from an approximation to Bayesian reasoning. Cognition 245:105693. doi: 10.1016/j.cognition.2023.105693, 38244398

[ref66] Ratcliff R. McKoon G. (2008). The diffusion decision model: Theory and data for two-choice decision tasks. Neural Comput. 20, 873–922. doi: 10.1162/neco.2008.12-06-420, 18085991 PMC2474742

[ref67] Rescorla R. Wagner A. (1972). “A theory of Pavlovian conditioning: Variations in the effectiveness of reinforcement and nonreinforcement,” in Classical Conditioning II: Current Research and Theory, eds. Black A. Prokasy W. (New York: Appleton Century Crofts), 64–99.

[ref68] Ross R. McKay R. Coltheart M. Langdon R. (2015). Jumping to conclusions about the beads task? A meta-analysis of delusional ideation and data-gathering. Schizophr. Bull. 41, 1183–1191. doi: 10.1093/schbul/sbu187, 25616503 PMC4535629

[ref69] Samuelson W. Zeckhauser R. (1988). Status quo bias in decision making. J. Risk Uncertain. 1, 7–59. doi: 10.1007/bf00055564

[ref70] Savage L. (1954). The Foundations of Statistics. New York: Wiley.

[ref71] Seer C. Lange F. Boos M. Dengler R. Kopp B. (2016). Prior probabilities modulate cortical surprise responses: a study of event-related potentials. Brain Cogn. 106, 78–89. doi: 10.1016/j.bandc.2016.04.011, 27266394

[ref72] Shenhav A. Botvinick M. Cohen J. (2013). The expected value of control: An integrative theory of anterior cingulate cortex function. Neuron 79, 217–240. doi: 10.1016/j.neuron.2013.07.007, 23889930 PMC3767969

[ref73] Simon H. (1956). Rational choice and the structure of the environment. Psychol. Rev. 63, 129–138. doi: 10.1037/h0042769, 13310708

[ref74] Stephan K. Mathys C. (2014). Computational approaches to psychiatry. Curr. Opin. Neurobiol. 25, 85–92. doi: 10.1016/j.conb.2013.12.007, 24709605

[ref75] Tenenbaum J. Kemp C. Griffiths T. Goodman N. (2011). How to grow a mind: statistics, structure, and abstraction. Science 331, 1279–1285. doi: 10.1126/science.1192788, 21393536

[ref76] Todd P. Gigerenzer G. (2007). Environments that make us smart: ecological rationality. Curr. Dir. Psychol. Sci. 16, 167–171. doi: 10.1111/j.1467-8721.2007.00497.x

[ref77] Tversky A. Kahneman D. (1973). Availability: a heuristic for judging frequency and probability. Cogn. Psychol. 5, 207–232. doi: 10.1016/0010-0285(73)90033-9

[ref78] Tversky A. Kahneman D. (1974). Judgment under uncertainty: heuristics and biases. Science 185, 1124–1131. doi: 10.1126/science.185.4157.1124, 17835457

[ref79] Tversky A. Kahneman D. (1992). Advances in prospect theory: cumulative representation of uncertainty. J. Risk Uncertain. 5, 297–323. doi: 10.1007/bf00122574

[ref80] Usher M. McClelland J. (2001). The time course of perceptual choice: The leaky, competing accumulator model. Psychol. Rev. 108, 550–592. doi: 10.1037/0033-295X.108.3.550, 11488378

[ref81] Visalli A. Capizzi M. Ambrosini E. Kopp B. Vallesi A. (2021). Electroencephalographic correlates of temporal Bayesian belief updating and surprise. NeuroImage 231:117867. doi: 10.1016/j.neuroimage.2021.117867, 33592246

[ref82] Visalli A. Capizzi M. Ambrosini E. Kopp B. Vallesi A. (2023). P3-like signatures of temporal predictions: a computational EEG study. Exp. Brain Res. 241, 1919–1930. doi: 10.1007/s00221-023-06656-z, 37354350

[ref83] von Helmholtz H. (1867). Handbuch der Physiologischen Optik. Leipzig: Voss.

[ref84] Wakker P. (2010). Prospect Theory: For Risk and Ambiguity. Cambridge, UK: Cambridge University Press.

[ref85] Wald A. (1947). Sequential Analysis. New York: Wiley.

[ref86] Zhang H. Maloney L. (2012). Ubiquitous log odds: a common representation of probability and frequency distortion in perception, action, and cognition. Front. Neurosci. 6:1. doi: 10.3389/fnins.2012.00001, 22294978 PMC3261445

